# Alterations in Neurogenic Inflammatory Responses in Mice Lacking αCGRP

**DOI:** 10.1100/tsw.2001.423

**Published:** 2001-12-19

**Authors:** Jongho Lee, Ronald B. Emeson

**Affiliations:** Department of Pharmacology, Vanderbilt University, Nashville, TN 37232-6600, USA

## Abstract

a-Calcitonin gene-related peptide (aCGRP) is a pleiotropic peptide neuromodulator that is widely expressed throughout the central and peripheral nervous systems. Although CGRP has been implicated in numerous physiological processes (including peripheral vasodilatation, acetylcholine receptor biosynthesis, nociception, and neurogenic inflammation), the precise physiological roles of CGRP remain to be elucidated. To provide a better understanding of the physiological role(s) mediated by this peptide neurotransmitter, we have generated aCGRP-null mice by targeted modification in embryonic stem cells.

